# Mid‐term comparison of new‐onset AHRE between His bundle and left bundle branch area pacing in patients with AV block

**DOI:** 10.1002/joa3.70009

**Published:** 2025-02-04

**Authors:** Catalin Pestrea, Ecaterina Cicala, Roxana Enache, Marcela Rusu, Radu Gavrilescu, Adrian Vaduva, Sever Risca, Dana Clapon, Florin Ortan

**Affiliations:** ^1^ Department of Interventional Cardiology Brasov County Clinical Emergency Hospital Brasov Romania

**Keywords:** atrial high‐rate episodes, His bundle pacing, left bundle branch area pacing, mid‐term follow‐up

## Abstract

**Background:**

Atrial high‐rate episodes (AHRE) detected by cardiac implanted electronic devices are known markers for adverse cardiac events. Previous studies have shown that the incidence of new‐onset AHREs in patients with right ventricular pacing reaches 50%. At the same time, His bundle pacing (HBP) and left bundle branch area pacing (LBBAP) were associated with significantly fewer AHRE. This study aimed to compare the incidence of new‐onset AHRE between HBP and LBBAP in patients with atrioventricular block and no history of atrial fibrillation.

**Methods:**

One hundred and forty‐two patients, fifty‐nine with HBP and eighty‐three with LBBAP for advanced atrioventricular block, were prospectively followed for new‐onset AHRE.

**Results:**

The mean follow‐up period was 624 ± 148.6 days for the HBP patients and 663.4 ± 157.4 days for the LBBAP patients. New‐onset AHRE was encountered in 8 of 59 patients (13.5%) with HBP and 14 of 83 (16.8%) with LBBAP (hazard ratio—0.91, log rank *p* = .84). In the multivariate Cox regression model, HBP and LBBAP had similar predictive values, while only age and diabetes mellitus were significantly associated with new‐onset AHRE occurrence.

**Conclusion:**

HBP and LBBAP were associated with a similar incidence of device‐detected new‐onset AHRE during a medium‐term follow‐up period in patients with atrioventricular block and no history of atrial fibrillation.

## Graphical abstract

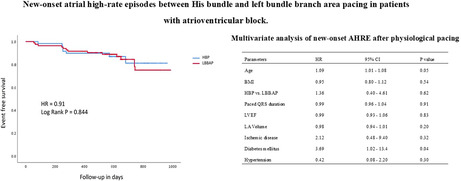

## 1 INTRODUCTION

Once conventional right ventricular myocardial pacing (RVP) became standard practice for patients with symptomatic bradycardic disorders, a new pathological entity called pacing‐induced cardiomyopathy (PICM) was introduced into clinical practice. With an incidence reaching up to 25% of the paced patients, PICM was initially defined as a reduction in the left ventricular ejection fraction (LVEF).^1^ However, the harmful effects of RVP extend beyond the impact on left ventricular (LV) performance, including worsening mitral regurgitation and atrial alterations.^2^, ^3^ The latter most often reflects an increased risk for atrial fibrillation (AF) development.^4^

As a marker of atrial myopathy, atrial high‐rate episodes (AHRE) are defined as atrial tachyarrhythmia episodes with rates >190 beats/min detected by cardiac implantable electronic devices and have a valuable role in evaluating the risk of symptomatic AF occurrence.^5^ Previous studies have shown that the incidence of new‐onset AHRE in patients with RVP reaches 50%, and they are associated with an increased risk of thromboembolic and major adverse cardiac events.^6^, ^7^

On the other hand, two physiological pacing techniques have been implemented in routine clinical practice to prevent and treat PICM: His bundle pacing (HBP) and left bundle branch area pacing (LBBAP). Both showed significant protective effects on LV function and significantly fewer AHRE than standard RVP.^8^, ^9^

This study compared the incidence of new‐onset AHRE between HBP and LBBAP in patients with atrioventricular block and no history of AF.

## 2 MATERIALS AND METHODS

### 2.1 Study design

This was a nonrandomized, prospective, single‐center study.

### 2.2 Patient selection

All consecutive patients with a successful physiological pacing procedure for second‐ or third‐degree atrioventricular block between June 2020 and December 2022 in the Cardiac Pacing Laboratory of the Brașov County Clinical Emergency Hospital in Romania were included prospectively in the local physiological pacing registry and eligible for enrollment in the present study. The inclusion criteria were as follows: (1) age over 18 years old; (2) proof of capture of the conduction system; (3) sinus rhythm with dual‐chamber pacemaker implantation; and (4) possibility to return for in‐person follow‐up visits. The exclusion criteria were as follows: (1) severe symptomatic valvular disease; (2) recent acute coronary syndrome or open‐heart surgery (within the previous 6 months); (3) A previous diagnosis of AF (irrespective of the type); (4) single‐chamber pacemaker implantation or biventricular pacing; and (5) life expectancy less than 1 year as a result of comorbidities.

In the end, 142 patients, 59 with HBP and 83 with LBBAP, qualified for analysis. The demographic and clinical characteristics were recorded at baseline. From the echocardiography examination, the LVEF was recorded using the biplane method of disks (modified Simpson's rule), and the left and right atrium volumes were recorded using the disk summation algorithms.

### 2.3 Pacing procedure

All conduction system pacing procedures used a C315 His or a C304 His catheter (Medtronic, Minneapolis, MN, USA) and a Select Secure 3830 lead (Medtronic).

The His bundle electrogram was initially identified for HBP, and the response to decremental pacing was assessed. A successful procedure was defined by a transition from nonselective to selective or myocardial capture. In patients with baseline bundle branch block, an additional correction (total or partial) of the bundle branch block was mandatory, with at least a 30% reduction in QRS duration.^10^

For LBBAP, the delivery kit was placed approximately 1.5 cm from the His bundle position toward the right ventricular apex, where the lead was screwed deep into the septum. To confirm the capture of the left‐sided conduction system, we used the criteria of a pseudo‐right bundle branch block morphology in lead V1 plus one of the following^11^:

- QRS transitions from nonselective to selective pacing with decremental or programmed stimulation.
- The presence of a left bundle branch/fascicular potential and a potential to R wave peak time equal to stimulus to R wave peak time.
- An LV activation time (measured as R‐wave peak time in lead V6) is shorter than 80 ms for a baseline narrow QRS complex and shorter than 90 ms for a wide‐baseline QRS complex.

No ventricular backup leads were implanted in all successful conduction system pacing cases. All patients received an atrial lead placed in the right atrial appendage. The leads were connected to a dual‐chamber pacemaker programmed in DDD mode at 60 bpm. The ventricular pacing minimization algorithms were turned off to ensure constant ventricular pacing. Also, all antitachycardia pacing functions were disabled. The targeted atrioventricular intervals for all patients were 120 ms for the atrial sensed value and 150 ms for the atrial paced value. In patients with LBBAP, the atrioventricular delays were programmed as such since the delay between the left bundle branch potential and QRS onset is negligible (usually less than 20 ms). In patients with HBP, the His‐to‐ventricle interval was subtracted from these values.

All procedural‐related parameters were recorded.

### 2.4 Follow‐up

The patients were followed in person in the outpatient clinic at 2, 6, and 12 months, and after that, every year. At each follow‐up, pacemaker interrogation was performed, and the pacing and sensing thresholds, the ventricular pacing burden, and the AHRE of more than 6 minutes at a rate ≥ 190 bpm (according to the 2017 ESC consensus on device detected subclinical atrial tachyarrhythmias) were recorded.^5^ All AHRE were manually reviewed for accuracy and to exclude noncardiac causes. Data collection was censored at the first AHRE occurrence or the end of the follow‐up period for those with no recorded AHRE.

### 2.5 Statistical analysis

Continuous variables were presented as mean ± 1 standard deviation, and categorical variables were presented as frequencies and percentages. The Shapiro–Wilk test was used to assess the normality of distribution. Statistical comparison of means was performed using the *t*‐test or the Mann–Whitney *U* test for independent groups and the *t*‐test or Wilcoxon test for dependent groups according to the normality of distribution. The Chi‐squared test evaluated the statistical difference between percentages. The Kaplan–Meier survival curve and the log‐rank test were used to estimate event‐free survival in the different pacing groups. Univariate Cox proportional hazards regression analysis was performed to investigate potential risk factors of postoperative new‐onset AHRE. Also, baseline variables considered to be clinically relevant or univariate predictors with *p* < .1 were entered into a multivariable Cox proportional hazard model.

For all tests, a two‐tailed *p* < .05 was considered statistically significant.

Statistical analysis was performed using SPSS software v 26.0 (IBM, Armonk, NY, USA).

### 2.6 Ethical considerations

The study was approved by the institutional ethics committee and complied with all aspects of the Declaration of Helsinki.

All patients were informed and provided their written consent before the procedure.

## 3 RESULTS

Table 1 presents the patients' baseline characteristics. The mean age was 67 ± 10.5 years, and the majority were men (68.3%). Patients in the LBBAP group were younger than those in the HBP group. The baseline QRS duration was similar between the two groups, as was the percentage of QRS morphologies. The overall LVEF was 50.3 ± 12, without significant differences between the two study groups. Also, there were no differences regarding comorbidities and medical treatment. None of the patients received class I or III antiarrhythmic drugs.

**TABLE 1:** Baseline patient characteristics.

	All	HBP	LBBAP	*p* value
Baseline characteristics				
Number of patients	142	59	83	
Age (years, mean ± SD)	67 ± 10.5	69 ± 8.5	65.5 ± 11.5	.04
Male (*N*, %)	97 (68.3)	38 (64.4)	59 (71.1)	.46
BMI (kg/m^2^, mean ± SD)	28.7 ± 3.9	28.5 ± 3.7	28.9 ± 4.2	.57
eGFR (mL/min, mean ± SD)	64.2 ± 17.7	65 ± 19.4	63.6 ± 16.4	.66
Baseline QRS				
QRS duration (ms, mean ± SD)	140.4 ± 34	136.2 ± 34	143.3 ± 34	.23
Normal QRS (*N*, %)	71 (50)	32 (54.2)	39 (47)	.49
LBBB (*N*, %)	52 (36.6)	20 (33.9)	32 (38.6)	.60
RBBB (*N*, %)	19 (13.4)	7 (11.9)	12 (14.5)	.80
Baseline echocardiography				
LVEF (%, mean ± SD)	50.3 ± 12	48.6 ± 12.4	51.4 ± 11.5	.15
LA volume (mL, mean ± SD)	62.5 ± 23.7	62.3 ± 18.4	62.6 ± 27.2	.94
RA volume (mL, mean ± SD)	40.9 ± 17.1	40.2 ± 14.2	41.5 ± 19.1	.71
Comorbidities				
Hypertension (*N*, %)	121 (85.2)	51 (86.4)	70 (84.3)	.81
Diabetes mellitus (*N*, %)	35 (24.6)	13 (22)	22 (26.5)	.56
Ischemic disease (*N*, %)	39 (27.5)	19 (32.2)	20 (24.1)	.34
Renal failure (*N*, %)	33 (23.2)	13 (22)	20 (24.1)	.84
Treatment				
RAAS antagonists (*N*, %)	102 (71.8)	41 (69.5)	61 (73.5)	.70
Beta‐blockers (*N*, %)	108 (76.1)	48 (81.4)	60 (72.3)	.24
MRAs (*N*, %)	42 (29.6)	18 (30.5)	24 (28.9)	.85

Abbreviations: BMI, body mass index; eGFR, estimated glomerular filtration rate; HBP, His bundle pacing; LA, left atrium; LBBAP, left bundle branch area pacing; LBBB, left bundle branch block; LVEF, left ventricular ejection fraction; MRAs, mineralocorticoid receptor antagonists; RA, right atrium; RAAS, renin–angiotensin–aldosterone system; RBBB, right bundle branch block; SD, standard deviation.

Table 2 shows the comparative procedural parameters. The paced QRS complex was significantly shorter in the HBP group than in the LBBAP group (119.1 ± 17.3 ms vs. 127.8 ± 16.3 ms, *p* = .003). On the other hand, LBBAP showed superiority for pacing and sensing thresholds. There was no difference in fluoroscopy and total procedural time.

**TABLE 2:** Comparative procedural characteristics between HBP and LBBAP.

Implant characteristics	HBP	LBBAP	*p* value
Number of patients	59	83	
Paced QRS duration (ms)	119.1 ± 17.3	127.8 ± 16.3	.003
Capture threshold (V)	0.9 ± 0.3	0.7 ± 0.3	.019
Capture threshold (ms)	1	0.4	
R‐wave detection (mV)	4.6 ± 2.5	9.8 ± 4.4	<.001
Pacing Impedance (Ohm)	460.6 ± 126.6	584.1 ± 146	<.001
Fluoroscopy time (min)	7.6 ± 4.9	8.2 ± 3.5	.368
Procedural time (min)	107.2 ± 27.2	116 ± 31.7	.09

Abbreviations: HBP, His bundle pacing; LBBAP, left bundle branch area pacing.

The mean follow‐up period was 624 ± 148.6 days for the HBP and 663.4 ± 157.4 days for the LBBAP group. During this period, the pacing threshold increased with HBP from 0.9 ± 0.3 to 1.1 ± 0.6 V at 1 ms pulse duration (*p* = .07) and decreased in the LBBAP group (0.7 ± 0.3 V vs. 0.6 ± 0.2 V at 0.4 ms pulse duration, *p* = .01).

As a result of the initial device programming, all patients had a more than 98% ventricular pacing burden. Table 3 presents all the follow‐up parameters.

**TABLE 3:** Comparative follow‐up parameters between HBP and LBBAP.

Follow‐up parameters	HBP	LBBAP	*p* value
Follow‐up duration (days)	624 ± 148.6	663.4 ± 157.4	.134
Capture threshold (V)	1.1 ± 0.6	0.6 ± 0.2	<.001
Capture threshold (ms)	1	0.4	
R‐wave detection (mV)	4.3 ± 2.6	11.9 ± 5.8	<.001

Abbreviations: HBP, His bundle pacing; LBBAP, left bundle branch area pacing.

**FIGURE 1:**
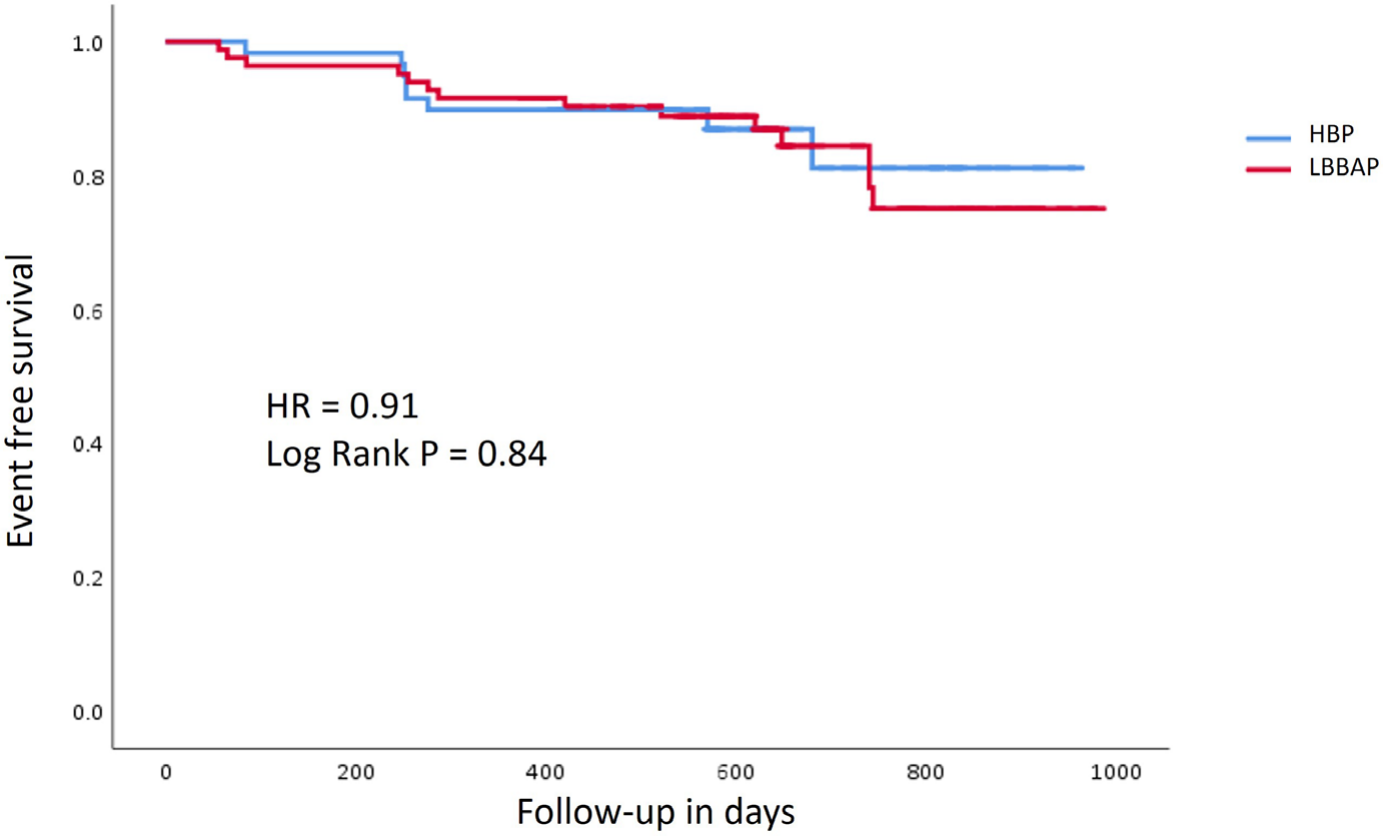
Kaplan–Maier curve for freedom from new‐onset AHRE between HBP and LBBAP. AHRE, atrial high‐rate episodes; HBP, His bundle pacing; HR, hazard ratio; LBBAP, left bundle branch area pacing.

Regarding the study's primary outcome, new‐onset AHREs were encountered in 8 of 59 patients (13.5%) with HBP and 14 of 83 (16.8%) with LBBAP. All eight patients in the HBP group had five or fewer AHREs, with a total AHRE burden of less than 1%. Twelve patients in the LBBAP group had five or fewer AHREs, with a total AHRE burden of less than 1%, and two patients had 23 and 27 AHREs with an AHRE burden of 3% and 4%, respectively. Figure 1 illustrates the nonsignificant difference in freedom from new‐onset AHRE between HBP and LBBAP during the follow‐up period (hazard ratio [HR]—0.91, log rank *p* = .84).

Table 4 presents the univariate analysis of baseline clinical features and potential predisposing factors for developing new‐onset AHRE. Only the presence of diabetes mellitus was significantly associated with an increased risk of new‐onset AHRE [HR 2.39 (1.03–5.58, 95% CI), *p* = .04]. None of the other factors, including the type of physiological pacing, were significantly associated with this outcome.

**TABLE 4:** Univariate analysis of new‐onset AHRE after physiological pacing.

Parameters	HR	95% CI	*p* value
Age	1.027	0.98–1.07	.24
Gender	0.75	0.29–1.93	.56
BMI	0.93	0.84–1.05	.24
eGFR	1.00	0.98–1.02	.90
HBP vs. LBBAP	0.91	0.38–2.19	.84
Baseline QRS duration	0.99	0.98–1.01	.35
Paced QRS duration	1.00	0.98–1.03	.80
LVEF	0.99	0.96–1.03	.67
LA volume	0.98	0.96–1.01	.27
RA volume	0.98	0.95–1.02	.45
Ischemic disease	1.55	0.62–3.85	.34
Diabetes mellitus	2.39	1.03–5.58	.04
Hypertension	0.44	0.17–1.13	.09

Abbreviations: BMI, body mass index; CI, confidence interval; eGFR, estimated glomerular filtration rate; HBP, His bundle pacing; HR, hazard ratio; LA, left atrium; LBBAP, left bundle branch area pacing; LVEF, left ventricular ejection fraction; RA, right atrium.

We also used a multivariate Cox regression model to evaluate the independent risk factors of new‐onset AHRE, as shown in Table 5. Variables with a *p*‐value of <.1 in univariate analysis and those we considered clinically relevant were entered into the multivariate regression model. In this setting, besides diabetes mellitus, age was also significantly associated with new‐onset AHRE occurrence. The type of physiological pacing and the baseline echocardiographic showed no statistical significance.

**TABLE 5:** Multivariate analysis of new‐onset AHRE after physiological pacing.

Parameters	HR	95% CI	*p* value
Age	1.09	1.01–1.08	.05
BMI	0.95	0.80–1.12	.54
HBP vs. LBBAP	1.36	0.40–4.61	.62
Paced QRS duration	0.99	0.96–1.04	.91
LVEF	0.99	0.93–1.06	.83
LA volume	0.98	0.94–1.01	.20
Ischemic disease	2.12	0.48–9.40	.32
Diabetes mellitus	3.69	1.02–13.4	.04
Hypertension	0.42	0.08–2.20	.30

Abbreviations: BMI, body mass index; CI, confidence interval; HBP, His bundle pacing; HR, hazard ratio; LA, left atrium; LBBAP, left bundle branch area pacing; LVEF, left ventricular ejection fraction.

## 4 DISCUSSION

The study's main finding was that HBP and LBBAP showed no significant difference regarding new‐onset AHRE in patients with atrioventricular block and no history of AF.

AHRE are considered precursors of AF and a marker of atrial cardiomyopathy. Previous studies have shown that RVP was significantly associated with a higher rate of new‐onset AHRE (up to 30%) in patients without previous AF.^12^ Although not definitively proven, the most likely explanation for this association is the adverse LV remodeling during chronic RVP as a result of inter‐ and intraventricular dyssynchrony. This leads to increased LV filling pressures and, consequently, increased atrial pressures with morphological and electrical atrial remodeling acting as a trigger for atrial arrhythmias.^13^

Based on this argument, the most plausible protective mechanism of physiological pacing is the long‐term protective effect on LV morphology and function. The common pathophysiological feature of HBP and LBBAP is the rapid activation of the left‐sided conduction system, leading to a fast and synchronous LV activation.^14^ The difference between the two techniques is that during HBP, both ventricles are activated simultaneously, thus making it the most physiological pacing method. At the same time, during LBBAP, there is a slight delay in right ventricle activation, which is responsible for the longer QRS duration observed. Nevertheless, when translated into clinical practice, both physiological pacing modalities showed significant benefits over RVP regarding heart failure hospitalizations, death, and upgrade to biventricular pacing.^15^, ^16^ Also, several studies have proven that HBP and LBBAP preserve the LVEF in patients with baseline normal values and significantly improve the LVEF in patients with baseline decreased values.^17^

In a study of 105 patients with LBBAP, Bednarek et al.^18^ showed that after a mean follow‐up of 20 months, none of the patients reached the criteria for PICM and that the atrial volumes and diastolic echocardiographic measurements were not significantly changed compared to the baseline values. This positive effect of physiological pacing on the atrial function could be responsible for the lower incidence of new‐onset AHRE in this population.

Several studies presented a head‐to‐head comparison between physiological pacing and RVP while looking at new‐onset atrial tachyarrhythmias. In one of the first studies conducted by Ravi et al.,^8^ HBP demonstrated a lower risk of new‐onset AF (adjusted hazard ratio [HR], 0.53; 95% CI, 0.28–0.99; *p* = .046) compared with traditional RVP among 148 patients (HBP, *n* = 72; RVP, *n* = 76) with no history of AF. This study noted a new diagnosis of AF in 20.8% of patients in the HBP group and 40.8% in the RVP group (*p* = .009). Also, Takahashi et al.^19^ showed a significantly lower incidence of new‐onset AHRE in the HBP group than in the right ventricular septal pacing group (11% vs. 43%, *p* = .01). On the other hand, Zhang et al.^20^ investigated 175 patients with atrioventricular block and proved that the incidence of new‐onset AHREs in the LBBAP group was lower than in the RVP group (19.8% vs. 34.7%; *p* = .04). On the same note, Yang et al.^21^ found that 12.5% of the LBBAP patients developed new AHRE episodes, significantly less than right ventricular apex (36.4%) and septal pacing (61.5%).

In our study, the incidence of new‐onset AHRE with either pacing method was similar to the above references. Notably, the ventricular pacing burden was variable in most of the presented studies. In our study, since all patients had advanced atrioventricular conduction abnormalities, the ventricular pacing burden was very high (close to 100%), which continuously exposed the patients to the protective effect of physiological pacing and a constant atrioventricular synchronization as a result of the programmed atrioventricular delay.

Another interesting finding was that none of the echocardiographic baseline characteristics were associated with the occurrence of AHRE. This was in line with previous published results, which identified only the pacing method (physiological pacing vs. RVP) as a strong predictor for AHRE occurrence.^22^ Since our study population consisted of consecutive patients with physiological pacing irrespective of their baseline LVEF or other echocardiographic characteristics, this could be a strong argument for the overall protective effects of physiological pacing against new‐onset AHRE in all patients. On the other hand, we showed that, in our study group, age and diabetes mellitus were strongly associated with new‐onset AHRE. This was unsurprising since both factors are well‐established predictors for AF occurrence.^23^

Also, from the procedural point of view, our results confirmed that the acute pacing threshold was significantly better with LBBAP and that the His bundle capture threshold increased during follow‐up, although nonstatistically significant. Also, both pacing procedures produced narrow QRS complexes with a superior advantage from HBP.^24^

To our knowledge, this was the first medium‐term prospective study to compare the risk of new‐onset AHRE between the two currently used physiological pacing techniques in clinical practice. Another advantage of the study was that we programmed all the devices to deliver continuous pacing to increase the strength of comparison between the two groups.

On the other hand, several limitations must be mentioned. First of all, this was a single‐center, prospective study. Although it included a decent number of patients for a physiological pacing study, the strength of the evidence is limited. Another limitation is the lack of a control RVP group. Nevertheless, since there have been a significant number of studies on the incidence of new‐onset AHRE with conventional pacing, which generated consistent results, we believe that not having such a control group does not significantly diminish the value of the results. Although all recorded AHRE episodes were manually checked, false‐positive results are always possible. Other studies used slightly different criteria for AHRE regarding heart rate and duration. Without precise data, we cannot state for certain, but we do not believe using other criteria would have significantly changed the results.

## 5 CONCLUSION

HBP and LBBAP were associated with a similar incidence of device‐detected new‐onset AHRE during a medium‐term follow‐up period in patients with atrioventricular block and no history of AF.

## AUTHOR CONTRIBUTIONS

Conceptualization: Catalin Pestrea, Ecaterina Cicala, Roxana Enache, Marcela Rusu, Radu Gavrilescu, Adrian Vaduva, Sever Risca, Dana Clapon, and Florin Ortan. Methodology: Catalin Pestrea, Ecaterina Cicala, Roxana Enache, Marcela Rusu, Radu Gavrilescu, Adrian Vaduva, Sever Risca, Dana Clapon, and Florin Ortan. Formal analysis and investigation: Catalin Pestrea, Ecaterina Cicala, Roxana Enache, Marcela Rusu, Radu Gavrilescu, Adrian Vaduva, Sever Risca, and Dana Clapon. Writing – Catalin Pestrea and Ecaterina Cicala. Writing, review, and editing—Catalin Pestrea, Ecaterina Cicala, and Florin Ortan. Resources: Catalin Pestrea, Ecaterina Cicala, Roxana Enache, Marcela Rusu, Radu Gavrilescu, Adrian Vaduva, Sever Risca, Dana Clapon, and Florin Ortan. Supervision: Catalin Pestrea and Florin Ortan.

## CONFLICT OF INTEREST STATEMENT

Dr. Catalin Pestrea has received proctorship fees and travel grants from Medtronic.

## ETHICS STATEMENT

Approval of the research protocol: The study was approved by the Ethics Committee of the “Iuliu Hațieganu” University of Medicine and Pharmacy in Cluj‐Napoca (Number AVZ34/14.02.2022) and the Ethics Committee of the Brasov County Emergency Clinical Hospital (Number 30/21.03.2022) and was conducted under all of the ethical principles of the Seventh Revision of the Helsinki Declaration from 2013.

Informed consent: All patients signed an informed consent before the pacing procedure and the inclusion in the study.

Registry and the Registration No: N/A.

Animal studies: N/A.

## Data Availability

The data included in this article are available upon reasonable request.
